# Driving Difficulties and Adaptive Strategies: The Perception of Individuals Having Sustained a Mild Traumatic Brain Injury

**DOI:** 10.1155/2012/837301

**Published:** 2012-02-13

**Authors:** Carolina Bottari, Marie-Pierre Lamothe, Nadia Gosselin, Isabelle Gélinas, Alain Ptito

**Affiliations:** ^1^Occupational Therapy Program, School of Rehabilitation, Université de Montréal, Montreal, QC, Canada H3C 3J7; ^2^Centre for Interdisciplinary Research in Rehabilitation of Greater Montreal, Montreal, QC, Canada H2H 2N8; ^3^Department of Neurology & Neurosurgery, McGill University, Montreal, QC, Canada H3A 2B4; ^4^Cognitive Neuroscience Unit, Montreal Neurological Institute and Hospital, Montreal, QC, Canada H3A 2B4; ^5^School of Physical & Occupational Therapy, McGill University, Montreal, QC, Canada H3G 1Y5; ^6^Department of Psychology, McGill University Health Centre, Montreal, QC, Canada H3A 1A1

## Abstract

*Introduction*. After a mild traumatic brain injury (mTBI), individuals quickly resume driving. However, relatively little is known about the impact of mTBI on driving ability and, notably, on the perceived influence of postconcussive symptoms on driving. Hence, the objective of this study was to document the perception of driving abilities in individuals with mTBI. *Method*. Twenty-seven drivers with mTBI were interviewed to document their perception regarding their driving abilities. Both driving-related difficulties and compensatory strategies used to increase driving safety were documented. A mixed quantitative and qualitative analysis of the data was completed. *Results*. 93% of participants reported at least one difficulty perceived as having an impact on everyday activities. Most frequently named problems affecting driving were fatigue and reduced concentration. In addition, 74% of participants had adapted their driving or developed strategies to compensate for driving difficulties. *Discussion/Conclusion*. Postconcussive symptoms have repercussions on driving ability. However, people with mTBI tend to be aware of their difficulties and develop, over time, adaptive strategies. Preventive measures are thus warranted to increase health care professionals' awareness of the potential consequences of mTBI on driving ability and to promote guidelines for the safe resumption of driving after injury.

## 1. Introduction

Mild traumatic brain injury (mTBI) is a major public health problem disproportionately affecting young adults [1]. Incidence in the United States is about 600 per 100,000 population, though many do not seek medical care, and only 25% are admitted to hospital [1]. It is estimated that up to 25% of individuals presenting to the emergency department with mTBI have persisting postconcussive symptoms at 6 months following injury [2], such as headaches, fatigue, concentration deficits, delayed information processing, and vision and memory problems. Despite these problems, individuals generally resume driving shortly after their mTBI. However, very few studies have examined the potential consequences of mTBI on driving ability to promote positive strategies and guidelines for the safe resumption of driving after injury. In the present study, this issue will be investigated by obtaining the perspective of individuals who have sustained a mTBI in order to explore and describe the problems they experience in relation to driving, and the strategies they utilize to ensure safety when driving.

Driving is a complex, cognitively demanding activity [3]. It requires planning, concentration, inhibition of nonpertinent stimuli, foresight, anticipation, problem solving, a capacity to interpret complex situations with multiple stimuli quickly and efficiently, and the capacity to react calmly, but rapidly and effectively [3]. For many people, driving is synonymous with independence [3], and, in most cases, persons who have experienced an injury wish to resume driving as quickly as possible [4]. However, the evaluation of driving is challenging for health care professionals, especially with the TBI clientele, since there are few standardized protocols of evaluation adapted to their needs [5, 6] and the efficiency of the existing measures is not well known [7].

Symptoms such as fatigue and difficulty concentrating are known contributors to “quasi accidents” (or near misses). Near misses are events that do not cause injury and have limited immediate impact [8]. However, near misses have been shown to be good indicators of drivers at higher risk of being implicated in accidents. If a person experiences drowsiness, near-misses are 14 times more common than actual accidents [8]. In terms of concentration, inattention of drivers plays a major role in road accidents for the general population. The National Highway Traffic Safety Administration (NHTSA) estimates that about 25% of accidents reported by the police involve a certain form of inattention of the driver—the driver is distracted, asleep or fatigued, or “lost in his/her thoughts” [9].

It has recently been shown that persons with mTBI are at a higher risk of collisions when driving [10]. Using a survey of on-road-related activities with a group of 80 college undergraduate students, Schneider and Gouvier found that persons who self-reported a history of mTBI also self-reported a significantly higher number of accidents than control subjects and were classified in a higher risk category for vehicular crashes [10]. Average age of participants was 22 years and average time following mTBI was 7.1 years. Preece et al. (2010) studied the risk incurred on driving for persons with mTBI in the first 24 hours after the trauma. Here, participants were recruited in the emergency department within 24 hours of their injury, had a Glasgow Coma Scale score between 13 and 15, and were between 18 and 65 years of age. Fifty-seven percent had sustained a mTBI secondary to an assault and 26% secondary to a fall. Using the Hazard Perception Test, a video simulation test of road scenes requiring participants to rapidly identify problematic situations (i.e., probable collisions), mTBI participants were significantly slower to react than participants having sustained an orthopaedic injury (450 ms slower) [4]. This slower reaction time would translate into an increased breaking distance of 7.50 m when travelling at a speed of 60 km/h. These results suggest an increased risk of accidents secondary to a mTBI in the first 24 hours; therefore abstaining from driving during this time is crucial to promote driving safety for this population [4].

Despite the potential consequences of mTBI on driving, few guidelines for return to driving have been published. This is surprising when one considers that driving is such a complex and high risk activity regarding potential injury to both self and others, seemingly as important as return to playing sports, where several clinical practice guidelines [11] ensure that athletes return to their sport at the safest time [12]. Considering the lack of clinical guidelines and the problems raised for clinical practice concerning the return to driving following a mTBI, the objectives of the study were to (1) document the perception of driving abilities in individuals having sustained a mTBI who remain symptomatic, (2) analyze the difficulties reported by individuals with mTBI in relation to their impact on driving, and (3) explore whether individuals with mTBI use compensatory strategies to facilitate safe driving.

## 2. Methodology

### 2.1. Study Design

A qualitative research method was used to attain the objectives of this study.

### 2.2. Participants

Twenty-seven drivers with postconcussive symptoms secondary to a mTBI participated in this study. Participants were recruited from the McGill University Health Centre and Hôpital du Sacré-Coeur de Montréal, two major tertiary trauma centers in the Montreal area, Canada, as part of a larger study on mTBI conducted by our research team. Potential participants with postconcussive symptoms were identified by their physicians and informed of our study. Those interested in the study were then contacted by the researchers. The inclusion criteria were the following: (a) diagnosis of mTBI confirmed by an expert physician, (b) presence of symptoms (regardless of the severity), and (c) between 18 and 65 years of age. Exclusion criteria included (a) litigation issues related to the TBI, (b) presence or history of neurological or psychiatric problems, and (c) substance abuse. The study was approved, as part of a broader study on the measure of complex everyday activities in individuals with mTBI, by the ethics committees of the McGill University Health Centre and the Centre for Interdisciplinary Research in Rehabilitation of Greater Montreal. All the participants signed a consent form.

### 2.3. Data Collection

The information was collected from participants using semistructured interviews based on the ADL Profile [13] and open-ended questions specific to driving. The ADL Profile is an evaluation tool used to document independence in everyday activities [13] that has well-documented psychometric properties for use with individuals with TBI [13–15]. The ADL Profile consists of 20 activities (six activities linked to personal care, five linked to domestic activities, and nine linked to community activities) [13]. Two methods of evaluation are included in the test: a performance-based measure of everyday activities and a questionnaire administered as a semi-structured interview. In the present study, only the portion of the semi-structured interview regarding independence in community activities was used, that is, in our case, independence in driving and self-perceived difficulties and their impact on everyday activities. Open-ended questions specific to driving covered the strategies used to overcome their driving difficulties and whether they felt safe when driving. With the consent of participants, 17 of the 27 interviews were recorded. These interviews were fully transcribed and the verbatim analyzed. Written notes taken during the interviews were used to analyze the results of those participants who refused to be video taped. Sociodemographic and clinical information were also recorded.

Participants also completed the following tests: the Post-Concussion Scale Revised (PCSR) [16, 17], the Beck Anxiety Inventory (BAI) [18], the Posttraumatic Stress Disorder Checklist (PCL-S) [19], the Fatigue Severity Scale (FSS) [20], and the Beck Depression Inventory, Second Edition (BDI-II) [21].

### 2.4. Data Analysis

Both quantitative and qualitative analyses were performed on the data. First, a qualitative analysis of the transcripts was completed using a content analysis [22]. All transcripts were read several times, highlighting the text that appeared most informative of the repercussions of mTBI on driving ability and writing keywords or phrases (*preliminary codes*) using the participants' words. After open coding of three or four transcripts, the preliminary codes were assigned to the pertinent excerpts related to the research questions for all remaining transcripts, and new concepts were added when data that did not fit into existing codes was encountered. Once all transcripts were coded, the data within a particular code was either regrouped or eliminated when codes were deemed nonpertinent. During the last stage, the theoretical model of car driving proposed by Michon [23] was used to complete the final analysis of the data and to frame the presentation of the results regarding the perception individuals with mTBI have of their driving difficulties and of the strategies they use to overcome them. Data analysis was completed by two researchers (C. Bottari and M-P. Lamothe) to ensure reliability of the coding and of the interpretation of the data. Second, a quantitative analysis was completed to obtain frequencies of observed themes over all participants and scores on the ADL Profile interview.

### 2.5. The Hierarchical Model of Task Performance in Car Driving

The Hierarchical Model of Task Performance in Car Driving [23, 24] is a theoretical model that is largely cited in the scientific literature related to driving [5, 25–28]. This model looks at decision making in three levels: *strategic* (planning), *tactical* (manoeuvring), and *operational* (control) level. The *strategic* level refers to decision making that occurs over long periods of time, prior to taking the wheel. It involves general planning of trip goals, route, modal choice and the evaluation of costs and risks associated with the trip. The *tactical* level of decision making occurs on the road and is influenced by environmental conditions. Here the driver exercises manoeuvre control which allows him or her to interact with the circumstances faced on the road. This level calls upon complex cognitive functions, flexibility, and awareness of the actual demands of the road. Manoeuvres can be adapted to the situation, for example, choice of speed, overtaking a vehicle, distance between vehicles, and so forth, and are derived from the original goals set in the *strategic* level. This level of decision making takes place much more rapidly than strategic decisions as they occur over the space of seconds. Finally, it is within the *operational* (basic skill) level that immediate reactions are required. At this level, the individuals use the baseline skills of steering, braking, and so forth to cope with threats or perceived danger. These decisions occur over the space of milliseconds and depend essentially on the speed of perception of stimuli and driving skills.

## 3. Results

Of the 27 participants with postconcussive symptoms who participated in the study, all were currently active drivers except for one individual who was not currently driving as he had not yet received medical permission to drive. The sociodemographic characteristics of the participants, as well as the time after mTBI, are summarized in Table 1. Time after injury varied between two weeks and six years. Sixty-seven percent had sustained a sport-related accident and 19% a motor vehicle accident. Fifty-one percent had returned to work or school full time and 26% remained on medical leave of absence. Fifty-six percent of participants reported moderate or severe postconcussive symptoms (PCSR score > 21), 56% fatigue problems (FSS score > 36), 30% moderate or severe depression (BDI score > 20), 15% moderate or severe anxiety (BAI score > 22), and 33% reported posttraumatic stress disorder (PCLS score > 44). The average time between the accident and the return to driving for all participants was 19.8 days, with a standard deviation of 20.8 days. For the participants who waited the longest, the delay (one or two months) was secondary to the medical advice they had received. Alternately, four participants clearly mentioned not having waited at all to return to driving; they therefore drove in the first 24 hours following their mTBI.

### 3.1. Difficulties and Consequences in Everyday Functioning

At the time of the interview with the ADL Profile, 25 of the 27 participants (93%) mentioned at least one difficulty that limited them in the realization of their everyday activities. The difficulties reported by the participants, as well as the number of participants who identified them as having an impact on their participation in their overall activities of daily living (ADL), are indicated in Table 2. It is important to note that only those likely to interfere specifically with driving, according to the prerequisite skills for driving identified by Rizzo and Kellison [3], are reported in this study. Among the most prevalent problems, “fatigue” was present in 78% of participants; “concentration problems” and “memory problems” present, respectively, in 74% and 63% of our sample. In a slightly smaller proportion, we found “anger easily” (56%), “anxiety” (52%), and “difficulty doing more than one thing at a time” (48%).

### 3.2. Analysis of Driving Difficulties according to Michon's Model (1979, 1985)

Table 3 illustrates the difficulties reported by the participants concerning their driving, while Table 4 presents an analysis of perceived driving difficulties according to the affected level of decision making using Michon's model of car driving [23, 24]. Difficulties affecting the *operational* level included vision problems, physical pain, and loss of automatic driving reflexes. Other issues such as fatigue, delayed response, dizziness, and concentration problems influenced both the *operational* and *tactical* levels of decision making. In the current study, participants reported that headaches, anxiety, decreased anticipation, memory problems, spatial orientation problems, and irritability influenced their *tactical* level of decision making during driving. Finally, one participant reported difficulties at the *strategic* level. This individual reported never questioning whether his physical and mental states were optimal for safe driving prior to getting behind the wheel of his car.

### 3.3. Strategies to Facilitate Safe Driving

Twenty of the 27 drivers (74%) reported having developed certain strategies or changed certain behaviors to compensate for their difficulties when driving. For the most part, strategies were developed only after having attempted a return to previous driving habits and having then becoming aware of unexpected driving difficulties and subsequent unsafe driving behaviors. These strategies were classified according to Michon's model of car driving [23, 24], depending on the decision-making level to which they belonged (see Table 5). 

At the *strategic* level, the most common strategy used (**n** = 8) was reducing the travel distance or time spent driving. Four participants reported self-assessing their current aptness to drive prior to driving and reducing or abstaining from driving in the evening. These strategies were aimed at overcoming significant fatigue, concentration problems or significant headaches that interfered with their ability to drive a car. To explain the use of avoidance as a strategy to adapt to severe fatigue problems, one participant said:

*And then, well, if one day when I'm tired because the night before, I admit, I did things that made me very tired, well you know, I'll maybe just not take the car because I'll decide that I do not have to take it that day.*

The third strategic-level strategy (not driving at night) compensated for fatigue problems and also addressed visual difficulties as some participants reported an increased difficulty with their visual acuity, especially in the evening. One participant reported:

*But I do not drive at night (…) because at night I have a… since my accident, I have a problem with my vision (…) it's like I see stars.*

At the *tactical* level, not conversing with other passengers while driving, changing drivers along the way, and taking breaks or naps along the way were identified as strategies by three participants. These strategies helped overcome problems related to fatigue and concentration. For example, in talking about avoiding having a conversation with passengers, one participant said:

*When I speak, I am less… a little like everyone; I am not very efficient at driving.*

Additional tactical-level strategies mentioned by the participants included reducing one's speed, decreasing risk taking behaviours, increasing the space between vehicles, avoiding traffic, or limiting the frequency of lane changes. Participants who reported using these strategies tended to also report high levels of anxiety and slower reaction times.

*When I drive, I am maybe 90%. The 10% I miss, it's about my reaction speed. But I drive slower than before (…) well, 25% more slowly. I compensate.*

Strategies specific to the *operational* level were not explicitly mentioned by the participants. However, it is important to understand that Michon's three-level model is hierarchical and that decisions made at the tactical and strategic levels compensate for problems at the operational level.

### 3.4. Level of Independence in Driving and Strategies That May Optimize Driving a Car Safely

During the interview, participants were asked to give their perception of their current level of independence in relation to driving, based on the four-point ordinal scale of independence of the ADL Profile (dependence, requires verbal and/or physical assistance, independence with difficulty, independence) (see Table 6). Ten of the 27 participants said they were independent, 15 said they were independent with difficulty, one needed physical or verbal assistance, and one reported being dependent.

Even though 10 participants reported being able to drive independently, five of these mentioned using strategies to overcome various difficulties when driving. Of these five, two reported being “more anxious while driving since their accident”; one was now overall “more cautious” when driving and the other “avoided driving in traffic all together.” Still within the group of five who used strategies, two had reduced their time of continuous driving due to fatigue and two used somewhat risky strategies that included driving with his/her left leg when pain in the right leg was too severe and stopping on the side of the road to have a nap when overcome by fatigue.

For the 15 participants who perceived themselves as being independent with difficulty for driving, all but two reported using at least one strategy to compensate for difficulties that influenced their driving. One participant failed to put necessary strategies into place despite the presence of significant difficulties affecting his driving, that is, dizziness and important fatigue problems. This participant mentioned not questioning his physical or mental state prior to taking the wheel.

One participant reported needing assistance to drive; he stated that he only drives in more suburban or rural areas and asks his partner to drive for him in the city. One participant reported being dependent for driving, as he had not yet received the medical authorization to return to driving due to his persistent symptoms which were at risk of interfering with his driving.

## 4. Discussion

This study was one of the first to document the impact of mTBI on driving based on the perception of individuals with postconcussive symptoms at an average of 15 months after injury. Numerous postconcussive symptoms were clearly perceived by participants as having a negative impact on their driving ability (e.g., fatigue, difficulty concentrating or problems with memory, vision problems or hearing problems, dizziness, headaches, fear, and/or anxiety, etc.). However, participants tended to be aware of their difficulties and had developed strategies over time, and likely by trial and error (though this is not the safest approach), to compensate, and this helped them feel safer while driving. Indeed, awareness of one's own ability is a crucial skill for driving and is regarded as an important indicator of safe or unsafe driving [29].

### 4.1. Strategies Utilized to Facilitate Safer Driving

Individuals may fail to anticipate the repercussions of mTBI on their driving ability, resume their activities as prior to their accident, and only realize the need to modify their habits once they are confronted with driving difficulties [30]. Though this manner of resuming everyday activities may have little risk for certain everyday activities secondary to a mTBI, this adaptation period may carry greater risks for driving.

Individuals having sustained a mTBI tend to be more aware of their deficits than individuals with moderate or severe TBI [30]. According to Lundqvist and Alinder [5], individuals who are aware of their cognitive capacity are able to cope with cognitive impairments at the tactical level of decision making, as was seen in the present study. Awareness of deficits helps with developing compensatory strategies. In the present study, a majority of participants reported using at least one compensatory strategy to overcome their driving difficulties. Moreover, a person who is aware of having difficulties in the *operational* and *tactical* levels of driving will take appropriate decisions at the *strategic* level [5]. For example, participants in this study made *strategic-*level decisions to avoid night-time driving or reduce the distance they travel. Examples of *tactical*-level decisions were to slow down, minimize lane changes, and allow for a larger distance between vehicles. Such use of adaptive strategies at the *tactical* and *strategic* levels positively influences *operational* level decisions and can reduce accident risk [25].

At present, persons with mTBI's risk of minor accidents are likely greatest during the “exploration period” that occurs when first resuming driving after the accident. At this time, the person may not yet be aware of the impact of the mTBI on his driving ability and hence not have identified the need to modify his driving habits in consideration of his new condition. Therefore, the use of preventive interventions such as education programs on the potential impact of mTBI on driving and on potential strategies to reduce risk of accidents provided early on after the injury would merit further exploration.

### 4.2. Return to Driving following mTBI

Information concerning the delay between the occurrence of mTBI and the return to driving is not always available (both in general and in our participants), but the disparity in delays in the participants from this study deserves attention. In our study, at least four participants resumed driving within the first 24 hours of their mTBI. According to a study by Preece et al. [4], persons who have had a mTBI and are within the first 24 hours after mTBI were significantly slower to react in dangerous situations than a control group. The participants with mTBI in the present study were therefore potentially at risk when driving a car during the first 24 hours following their injury. Even though there are strict guidelines for return to play subsequent to mTBI, owing to the risks of re-injury and the aggravation of the symptomotology in athletes [31, 32], similar guidelines have not been well established for return to driving. Our results would suggest that persons having sustained, or suspected of having sustained, a mTBI should be informed that cognitive overload, which may occur secondary to a very rapid return to driving, can lead to an aggravation or a relapse of postconcussive symptoms even in individuals with mTBI who reported few symptoms [33]. Considering the safety concerns for the person with mTBI and society at large, strict guidelines to ensure the safe return to driving would need to be established with this population.

### 4.3. Limits to the Study

The aim of our study was to document the perception of driving abilities in individuals having sustained a mTBI who remain symptomatic. Our recruitment strategy specifically targeted symptomatic individuals which limits the generalization of these findings to non symptomatic individuals. The vast majority of individuals with mTBI are however likely to experience postconcussive symptoms within the first few days or weeks following their injury and equally likely to resume driving. Hence, results of the present study may generalize to acute mTBI and provide helpful insights to begin to improve clinical interventions in relation to mTBI and driving.

## 5. Conclusion

The principle objective of this study was to investigate the impact of mTBI on the resumption of driving from the perception of symptomatic and currently active mTBI drivers. It aimed to better understand the driving difficulties encountered by this population and the strategies used to compensate for the presence of these difficulties, the latter being an element of crucial importance to driving safety. It therefore provides a unique perspective to research related to the impact of mTBI on driving. Our results have demonstrated that persons with postconcussive symptoms report difficulties that slightly reduce their perceived level of independence in driving, as well as potentially increase the risk of minor driving accidents in individuals who fail to modify their habits to adapt to the presence of postconcussive symptoms. Nevertheless, they do report, for the most part, having developed strategies, over time and by trial and error (which is not the safest approach) for overcoming these difficulties. The risk of accidents is likely to be more elevated during the period where they try to resume driving while their strategies are not yet in place. We think that effective support of this clientele, by taking preventive actions and promoting positive driving strategies and guidelines for the safe resumption of driving after injury, could reduce the temporary safety risk. Additionally, further investigation into the consequences of mTBI on driving would be warranted to determine more effective methods of detecting overall risk when driving and readiness to resume driving. Knowledge translation strategies would also need to be identified to increase health care professionals' awareness of the potential consequences of mTBI on driving ability and to promote positive strategies and guidelines for the safe resumption of driving after injury.

## Figures and Tables

**Table 1:** Demographic and clinical profile of participants.

Characteristics	Frequency (%)
Total	27 (100)
Women	13 (48)
	Mean (SD)
Demographics	
Age	32,15 (10,35)
Years of education	14,39 (3,16)
Time after TBI (months)	14,59 (19,08)
Test results	
Postconcussion Symptom Scale Revised	40,89 (20,58)
Beck Anxiety Inventory	12,37 (7,79)
Posttraumatic Stress Disorder Checklist	39,04 (13,17)
Fatigue Severity Scale	38,93 (15,58)
Beck Depression Inventory, Second Edition	15,67 (10,34)

**Table 2:** Difficulties mentioned by the participants that have an impact on overall everyday activities.

Difficulties	*n*	%
Fatigue	21	77.78
Concentration	20	74.07
Memory	17	62.96
Anger easily	15	55.56
Fear, anxiety	14	51.85
Difficulty doing more than one thing at a time	13	48.15
Vision	10	37.04
Organisation	7	25.93
Headache	6	22.22
Hearing	5	18.52
Dizziness	3	11.11
Pain	2	7.41
Spatial orientation	2	7.41

**Table 3:** Examples of difficulties reported by the participants and their perceived repercussions on driving.

Difficulties	Verbatim examples that characterize the difficulties reported by the participants when driving
Fatigue	*I become …more tired a lot faster than before…*(Participant no. 300)

Vision problems	*Because at night I have a…since my accident, I have a problem with vision. (…) it's like I see stars*. (Participant no. 325)

Slowed reactions	*When I drive, I am maybe at 90%. (I'm missing 10%). It's due to my reaction speed. *(Participant no. 327)

Headaches	Evaluator*: You did not drive today because of your headache? * Participant*: Yes, I do not trust myself. *(Participant no. 314)

Anxiety	*I'm more stressed now about driving than before. *(Participant no. 323)

Loss of driving reflexes	*I loss the reflex of turning my head. (…) To check my blind spot, make the… good, you know: ≪OK, it's necessary that I check my blind spot… here… here… go!≫ *(Participant no. 329)

Decreased anticipation	*When driving, taking an exit, often I take it at the last minute. People must find me stupid. *(Participant no. 308)

Dizziness	*It was more difficult, even at the beginning. I did not drive at all because I was really dizzy during the first days. *(Participant no. 334)

Memory problems	*Sometimes I just simply forget where I'm heading. *(Participant no. 311)

Concentration problems	*It's like I forgot some of what happened on the road. Like I was elsewhere, but it was as if I was not really there and I do not remember what happened. *(Participant no. 318)

Spatial orientation problems	*You know, remember where I turned, I am not able to… like my visual memory of places, I do not have it anymore. In effect, it's my sense of orientation. *(Participant no. 323)

Angers easily	*The traffic, I always had a hard time with that and it did not get better with time but what I realized, is that I am more irritable since I had this accident. *(Participant no. 324)

Pain	*I have a lot of fatigue when driving long distances, with my leg. At a given moment, I have to remove my leg and change to drive with my left leg because I have fatigue and pain in my leg.* (Participant no. 336)

Difficulty remaining in driving lane	*(…) I keep looking behind me because I have a feeling that someone is going to hit me from behind, just like in my accident…I avoided driving on highways… I had a lot of difficulty staying in my lane. But after, it did not really get better like it was before. *(Participant no. 328)

Questions his/her ability to drive	* I do not really ask myself whether I'm able to drive or not. *(Participant no. 311)

**Table 4:** Analysis of perceived driving difficulties according to the affected level of decision making based on Michon's model of car driving (1979, 1985).

Level of decision according to Michon's Model (1979)	Strategic	Tactical	Operational
Difficulties			
Fatigue		X	X
Vision problems			X
Slowed reactions		X	X
Headaches		X	
Anxiety		X	
Loss of driving reflexes			X
Decreased anticipation		X	
Dizziness		X	X
Memory problems		X	
Concentration problems		X	X
Spatial orientation problems		X	
Angers easily		X	
Pain			X
Difficulty staying in his own driving lane			X
Questions his/her ability to drive	X		

**Table 5:** Compensation strategies identified by participants.

Level of decision according to Michon's Model (1979)	Examples of compensatory strategies	*n*	%
Strategic-level strategies (before taking the wheel)	Avoiding driving due to significant fatigue or headaches	4	14.81
	Reducing the distance travelled or time of continuous driving	8	29.63
	Reduction/abstaining from driving at night	4	14.81
	Avoiding rush hour	2	7.41
	Using a GPS	2	7.41

Tactical-level strategies (on the road)	Avoiding conversations while driving	2	7.41
	Taking breaks while driving or naps before driving	2	7.41
	Changing drivers along the way	3	11.11
	Reducing one's speed	5	18.52
	Being more careful, taking fewer risks	4	14.81
	Minimizing lane changes	1	3.70
	Allowing more distance between vehicles	1	3.70

**Table 6:** Perceived level of independence of participants for driving and the use of compensation strategies.

ADL profile scoring	*n*	%	Compensation strategies
3: independent without difficulty	10	37.04	Without: 4 (40%)
			With: 6 (60%)

2: independent with difficulty	15	55.56	Without: 2 (13, 33%)
			With: 13 (86,67%)

1v/p: verbal and/or physical assistance	1	3.70	With: 1 (100%)

0: Dependant	1	3.70	N/A (does not have medical authorization to drive)
